# Azilsartan Improves Salt Sensitivity by Modulating the Proximal Tubular Na^+^-H^+^ Exchanger-3 in Mice

**DOI:** 10.1371/journal.pone.0147786

**Published:** 2016-01-25

**Authors:** Masaki Hatanaka, Jun-Ya Kaimori, Satoko Yamamoto, Isao Matsui, Takayuki Hamano, Yoshitsugu Takabatake, Carolyn M. Ecelbarger, Shiro Takahara, Yoshitaka Isaka, Hiromi Rakugi

**Affiliations:** 1 Department of Geriatric Medicine and Nephrology, Osaka University Graduate School of Medicine, Suita, Osaka, Japan; 2 Department of Advanced Technology for Transplantation, Osaka University Graduate School of Medicine, Suita, Osaka, Japan; 3 Department of Comprehensive Kidney Disease Research, Osaka University Graduate School of Medicine, Suita, Osaka, Japan; 4 Department of Medicine, Division of Endocrinology and Metabolism, Georgetown University, Washington D.C., United States of America; George Washington University School of Medicine and Health Sciences, UNITED STATES

## Abstract

A potent angiotensin II type-1 receptor blocker, azilsartan, has been reported to reduce blood pressure more effectively than candesartan. Interestingly, azilsartan can also restore the circadian rhythm of blood pressure. We hypothesized that azilsartan could also improve salt sensitivity; thus, we examined the effect of azilsartan on sodium handling in renal tubules. Subtotal nephrectomized C57BL/6 mice received azilsartan (1.0 mg/kg/day), candesartan (0.3 mg/kg/day), or vehicle *via* the oral route in conjunction with a normal- (0.3%) or high-salt (8.0%) diet. Two weeks later, the azilsartan group showed significantly lower blood pressure during the light period than the candesartan and vehicle groups (azilsartan: 103.1 ± 1.0; candesartan: 111.7 ± 2.7; vehicle: 125.5 ± 2.5 mmHg; *P* < 0.05; azilsartan or candesartan *vs*. vehicle). The azilsartan group also showed higher urinary fractional excretion of sodium during the dark period than the candesartan and vehicle groups (azilsartan: 21.37 ± 3.69%; candesartan: 14.17 ± 1.42%; vehicle: 13.85 ± 5.30%; *P* < 0.05 azilsartan *vs*. candesartan or vehicle). A pressure—natriuresis curve demonstrated that azilsartan treatment restored salt sensitivity. Immunofluorescence and western blotting showed lower levels of Na^+^-H^+^ exchanger-3 (NHE3) protein (the major sodium transporter in renal proximal tubules) in the azilsartan group, but not in the candesartan or vehicle groups. However, azilsartan did not affect NHE3 transcription levels. Interestingly, we did not observe increased expression of downstream sodium transporters, which would have compensated for the increased flow of sodium and water due to non-absorption by NHE3. We also confirmed the mechanism stated above using cultured opossum kidney proximal tubular cells. Results revealed that a proteasomal inhibitor (but not a lysosomal inhibitor) blocked the azilsartan-induced decrease in NHE3 protein expression, suggesting that azilsartan increases NHE3 ubiquitination. In conclusion, azilsartan (but not candesartan) improved salt sensitivity possibly by decreasing NHE3 expression *via* ubiquitin—proteasomal degradation.

## Introduction

Hypertension is an important risk factor for cardiovascular and renal diseases. It is particularly evident in countries with a “westernized” lifestyle, in which ≈25% of the adult population is affected by hypertension [1].

Sodium intake has been demonstrated to be a modifiable cause of hypertension, which can lead to undesirable cardiovascular and renal outcomes [2]. Salt-sensitive individuals (normotensive and hypertensive) exhibit variable blood pressure levels after salt loading or restriction. Salt-sensitive patients are more prone to cardiovascular events and renal events than non-salt-sensitive hypertensive patients [3]. Furthermore, disturbances in the circadian rhythm of blood pressure (an independent predictor of cardiovascular events [4–9]) are closely associated with sensitivity to salt. Indeed, “non-dipper” hypertensive patients (i.e., those whose blood pressure does not decrease during the night) are more likely to exhibit salt sensitivity [10, 11].

Genes encoding sodium channels and sodium transporters in the kidney are known to be associated with salt sensitivity. Na^+^-K^+^-Cl^−^ cotransporter-2 (NKCC2) has been implicated in salt-sensitivity in the rat Milan hypertensive strain of rats [12]. The α2-adrenergic receptor, WNK lysine-deficient protein kinase-4, and Na^+^-Cl^−^ cotransporter (NCC) have been shown to be involved in the development of salt-sensitive hypertension in C57BL/6 mice and Dahl rats [13]. However, little attention has been paid to the sodium exchanger Na^+^-H^+^ exchanger-3 (NHE3), which is expressed in proximal tubules as a regulator of salt sensitivity [14].

Renin—angiotensin system (RAS) blockers are a mainstay of antihypertensive therapy for protection against hypertensive-based organ damage [15, 16]. However, RAS blockers have been judged to be unfavorable for the treatment of salt-sensitive hypertension. Indeed, the antihypertensive effects of RAS blockers are canceled out under high salt loading in hypertensive patients [17] and in animal models of hypertension [18, 19]. RAS blockers have even been reported to enhance salt sensitivity [20, 21]. However, recent studies in hypertensive patients have demonstrated that treatment with the novel angiotensin receptor blocker (ARB) azilsartan persistently lowers blood pressure over a 24-h period compared with other ARBs, and improves nocturnal hypertension more effectively than candesartan [22–24], suggesting that azilsartan has potential to restore the circadian rhythm of blood pressure.

In the present study, results showed that azilsartan improved salt-sensitive hypertension by enhancing NHE3 protein degradation through increased ubiquitination of the target protein.

## Materials and Methods

### Experimental animals

All procedures were carried out in accordance with guidelines for animal research set by the Animal Research Committee of Osaka University (approval number: DOI 24-016-001; Osaka, Japan).

Six-week-old male C57BL/6 mice were purchased from Japan SLC (Shizuoka, Japan). All mice were housed in an animal facility with a 12-h light—dark cycle and were provided water *ad libitum*. Anesthesia was induced using a combination of butorphanol tartrate (5.0 mg/kg, i.p.; Meiji Seika Pharma Co., Ltd., Tokyo, Japan), midazolam (4.0 mg/kg, i.p.; Astellas, Tokyo, Japan), and medetomidine (0.3 mg/kg, i.p.; Nippon Zenyaku Kogyo Co., Ltd., Fukushima, Japan).

Five-sixths nephrectomized (Nx) mice were generated by dissecting two-thirds of the left kidney mass and subsequent right unilateral nephrectomy after 7–10 days, as previously described [25]. After 5/6 Nx, blood pressure was measured during the light period every week using the tail-cuff method and a non-invasive blood pressure monitor (BP-98A, Softron Co., Ltd., Tokyo, Japan) as previously described [26, 27]. These measurements were not based on sensing blood flow in the tail, but on external detection of a tail pulse [28]. It has been confirmed that this method is compatible with direct intra-arterial recording of blood pressure in conscious mice [29]. Six weeks after 5/6 Nx, mice were randomized into nine groups. Three groups received a high-salt diet (8% NaCl), three groups received a medium-salt diet (4% NaCl; Oriental Yeast Co., Ltd., Tokyo, Japan), and the remaining three groups received a normal-salt diet (0.3% NaCl). Two weeks later, each of the groups was administered azilsartan, candesartan cilexetil, or vehicle. Azilsartan and candesartan cilexetil were resuspended in 0.5% methylcellulose (Wako Pure Chemical Industries, Osaka, Japan) at 1.0 mg/kg and 0.3 mg/kg, respectively, and administered by gastric gavage once daily. Doses of the two anti-hypertensive drugs were determined according to the highest doses used in humans (40 mg/day for azilsartan and 12 mg/day for candesartan). Azilsartan, candesartan cilexetil (for oral administration) and Candesartan (for in vitro studies) were provided by Takeda Pharmaceutical Company Ltd. (Tokyo, Japan). This fact does not alter our adherence to *PLOS ONE* policies on sharing data and materials.

After 2 weeks of treatment, all mice were housed without acclimatization in metabolic cages for 24 h to collect urine, and to measure the volume of urine produced and water consumed. These “tailor-made” cages were designed very carefully to prevent contamination of urine by feces or the high-sodium diet upon urine collection. Twelve-hour urine samples for each light and dark period were collected separately. Sodium measurements in urine were performed at SRL Inc. (Tokyo, Japan). Creatinine levels in urine were measured using an Aqua-Auto Kainos Cre-III Plus kit (Kainos Laboratories, Inc., Tokyo, Japan).

Mice were decapitated and arterial blood was immediately collected from the common carotid artery. Serum was separated by centrifugation and stored at −80°C until further use. Serum levels of sodium, creatinine, and urea nitrogen were measured using the VetScan VS2 (Abaxis, Union City, CA, USA). Sodium concentrations in urine and blood were used to calculate the urinary sodium excretion volume (U_Na_V) and fractional excretion of sodium (FENa). Remnant kidneys were harvested and divided into two parts. One part was immersed in 4% paraformaldehyde phosphate-buffered saline (PBS; Wako), soaked for several hours in 20% sucrose in PBS, and embedded in Tissue-Tek^®^ OCT Compound (Sakura Finetechnical Co., Ltd, Tokyo, Japan) for immunofluorescence staining. The other part was immediately frozen in liquid nitrogen and then subjected to extraction of total RNA. Additionally, proteins from the entire kidney, as well as proteins from proximal tubular brush border membranes, were extracted.

### RNA extraction, cDNA preparation, and reverse transcription-polymerase chain reaction (RT-PCR)

Total RNA was extracted from kidney tissue using TRIzol^®^ Reagent (Invitrogen, Carlsbad, CA, USA) according to manufacturer instructions and was reverse-transcribed successively using oligo (dT) and reverse transcriptase (PrimeScript RT Reagent kit, TakaraBio Inc., Shiga, Japan). Real-time quantitative PCR was done using SYBR Green (Applied Biosystems, Foster City, CA, USA) according to manufacturer instructions. Primers (forward and reverse, respectively) were as follows: mouse NHE3 (NM_001081060 XM_127434): GCTGTCATTGGCACTATATGG (424–444) and GAGGACTTCATTGACATGGAC (582–612); mouse β-actin (NM_007393): AGAGGGAAATCGTGCGTGAC (723–732) and CAATAGTGATGACCTGGCCGT (840–860); opossum NHE3 (L42522): CCACGAGCTCAACCTGAAG (394–377) and GACTGAGGCTTCTACAGTAGATGGACG (2004–2020); opossum β-actin (XM_001363445): GTGATCACCATTGGCAATGAGAG (591–613) and CGGTATTGGCATACAAATCCTTACG (991–1015). β-actin was used as a housekeeping gene.

### Protein extraction

Membrane vesicles from renal proximal tubular brush borders were prepared by Mg^2+^ aggregation, as previously described [30]. Briefly, dissected kidney cortices were homogenized in buffer A (300 mmol/l D-mannitol, 5 mmol/l ethylene glycol tetra-acetic acid, 12 mmol/l Tris-base; pH 7.1) at 4°C with an homogenizer (Polytron; Kinematica, Lucerne, Switzerland). MgCl_2_ was added to homogenates to a final concentration of 12 mmol/l. Homogenates were centrifuged at 1,500 × *g* for 15 min at 4°C. Supernatants were centrifuged at 25,000 × *g* for 30 min at 4°C, and membranes were pelleted from the final supernatants. Pellets were resuspended in buffer A, and protein content was assessed using the bicinchoninic acid method.

Whole kidneys were homogenized in Cell Lysis Buffer (Cell Signaling Technology Japan, K.K., Tokyo, Japan) at 4°C with a Polytron Homogenizer (Kinematica), centrifuged at 8,000 × *g* for 15 min at 4°C, and then supernatants were stored at −80°C until further use.

### Cell culture

Opossum kidney (OK) proximal tubular cells (American Type Culture Collection, Manassas, VA, USA) were cultured at 37°C in an atmosphere of 95% air/5% CO_2_ in Dulbecco’s modified Eagle’s medium with 10% fetal bovine serum, penicillin (100 U/ml), and streptomycin (100 mg/ml). Once confluent, the OK cells were treated with azilsartan (1.0 × 10^−6^ mol/l), candesartan (3.0 × 10^−7^ mol/l), or vehicle for 24 h, and simultaneously with angiotensin II (1.0 × 10^−11^ mol/l).

To evaluate the contributions of different protein-degradation routes, the effect of azilsartan on NHE3 was examined in the presence of inhibitors of lysosomal (leupeptin 0.5 μg/ml) (Wako) or proteasomal (lactacystin 10 μmol/l) (Wako) pathways of degradation. To assess the involvement of the angiotensin II type-2 receptor (AT2R), the AT2R antagonist PD123319 (10 μmol/l; (Wako) was used.

### Immunoblot analyses

Total cell lysates, whole-kidney homogenates, and extracted protein from renal proximal tubular brush border membranes were heated at 80°C for 2 min with an equal amount of Laemmli Sample Buffer (Bio-Rad, Hercules, CA, USA) containing 5% 2-mercaptethanol. They were resolved *via* 7.5% sodium dodecyl sulfate—polyacrylamide gel electrophoresis, and electrotransferred onto nitrocellulose membranes. After blocking with 5% skimmed milk and 0.1% Tween-20 in Tris-buffered saline (TBS) for 1 h, the blots were probed in the same buffer overnight with a mouse monoclonal anti-NHE3 antibody at 1:3000 dilution (clone 3H3), which was kindly provided by Dr. Peter Aronson (Yale University, CT, USA), rabbit polyclonal anti-NKCC2 antibody (Sigma—Aldrich Japan, Tokyo, Japan) at 1:1,000 dilution, anti-NCC antibody (Millipore, Temecula, CA, USA) at 1:1,000 dilution, anti-epithelial Na^+^ channel β (βENaC) [31] at 1:1,000 dilution, anti-β-actin antibody (Sigma—Aldrich Japan) at 1:5,000 dilution, or anti-glyceraldehyde 3-phosphate dehydrogenase (Sigma—Aldrich Japan) at 1:5,000 dilution. Blots were washed three times in 0.1% Tween-20 in TBS for 5 min, incubated with a 0.1 μg/ml horseradish peroxidase (HRP)-labeled goat anti-mouse immunoglobulin G (IgG) (Dako Japan, Tokyo, Japan) or 2.5 × 10^−2^ μg/ml HRP-labeled goat anti-rabbit IgG (Dako Japan) in 5% skimmed milk in 0.1% Tween-20 in TBS for 1 h, and washed as described above. Immunoreactive bands were visualized with chemiluminescence *via* ImageQuant LAS 4000 (GE Healthcare Japan, Tokyo, Japan) or CEPROS SV (Fujifilm, Tokyo, Japan). Band densitometry was carried out with ImageJ (National Institutes of Health, Bethesda, MD, USA).

### Immunoprecipitation

To measure the ubiquitination of total cellular NHE3, OK cells (60–80% confluent) were transiently transfected with a hemagglutinin (HA)-tagged ubiquitin (HA-Ub) expression vector, then treated with azilsartan (1.0 × 10^−6^ mol/l), candesartan (3.0 × 10^−7^ mol/l), or vehicle for 24 h, as described above. The cells were then lysed with ice-cold Cell Lysis Buffer for 1 h and centrifuged at 14,000 × *g* for 10 min at 4°C. The supernatants were incubated with anti-HA tag monoclonal antibody magnetic beads (MBL Co., Ltd., Aichi, Japan) at 4°C overnight. Immunoprecipitates were washed three times with PermaFluor Aqueous Mounting Medium. Pellets were resuspended in Cell Lysis Buffer and an equal amount of Laemmli Sample Buffer with 2-mercaptethanol (5%), heated for 2 min at 80°C, and subjected to immunoblotting with NHE3 antibody.

### Immunohistochemistry

Fixed and frozen remnant mouse kidneys were sectioned (thickness, 10 μm) using a cryostat (CM3050S; Leica Japan, Tokyo, Japan). Cultured OK cells on the coverslips were fixed in 4% paraformaldehyde PBS for 5 min. Sectioned kidney tissues and cultured cells were incubated overnight at 4°C with primary antibodies [a rabbit polyclonal anti-NHE3 antibody (Sigma-Aldrich Japan) at 1:200 dilution, anti-NKCC2 antibody at 1: 200 dilution, anti-NCC antibody at 1:500 dilution, βENaC at 1:400 dilution, and rabbit anti-ubiquitin antibody (Dako Japan, Tokyo, Japan) at 1:100 dilution. After washing three times with PBS, the samples were incubated at room temperature with secondary antibodies (Alexa Fluor 488-conjugated anti-mouse IgG antibody and/or Alexa Fluor 555-conjugated anti-rabbit IgG antibody, both at 1:400 dilution) plus 4′,6-diamidino-2-phenylindole dihydrochloride for 1 h. A drop of PermaFluor Aqueous Mounting Medium (Thermo Fisher Scientific, Waltham, MA, USA) was applied on sections or under cells. After mounting coverslips, the samples were observed using a confocal laser microscope (FV1000-D; Olympus, Tokyo, Japan). Characterization of antibodies against sodium transporters are summarized in S1 Table.

### Pulse-chase experiments

We conducted pulse-chase experiments for analyses of NHE3 protein in cultured OK cells after addition of azilsartan, as previously described [32]. Confluent cells were serum-deprived for 12 h and incubated twice without methionine or cysteine for 30 min. Cells were pulsed with 0.2 mCi/ml of ^35^S-methionine (Perkin—Elmer, Waltham, MA, USA) in methionine/cysteine-free medium (Thermo Fisher Scientific) for 16 h. After washing several times with PBS, cells were returned to the normal medium with angiotensin II and azilsartan, as described above. At 0, 12, 24, and 36 h, cells were harvested and underwent immunoprecipitation procedures using anti-NHE3 antibody. After 12 h of immunoprecipitation and washing three times with Cell Lysis Buffer, the immunoprecipitated samples were dissolved in Laemmli Sample Buffer and resolved on 7.5% sodium dodecyl sulfate polyacrylamide gels. After the gels had dried, radioactive NHE3 protein was quantified by autoradiography.

### Statistical analyses

Data are reported as mean ± standard deviation. Significance of differences was assessed by analysis of variance (ANOVA) with the *post-hoc* Tukey—Kramer test. In all tests, differences were considered significant at *P* < 0.05. Using the linear regression model, *P* < 0.10 indicated significance [33, 34]. Statistical analyses were carried out using JMP Pro 10 (SAS Institute Inc., Cary, NC, USA).

## Results

### Azilsartan significantly decreased blood pressure in salt-sensitive mice

**Fig 1:**
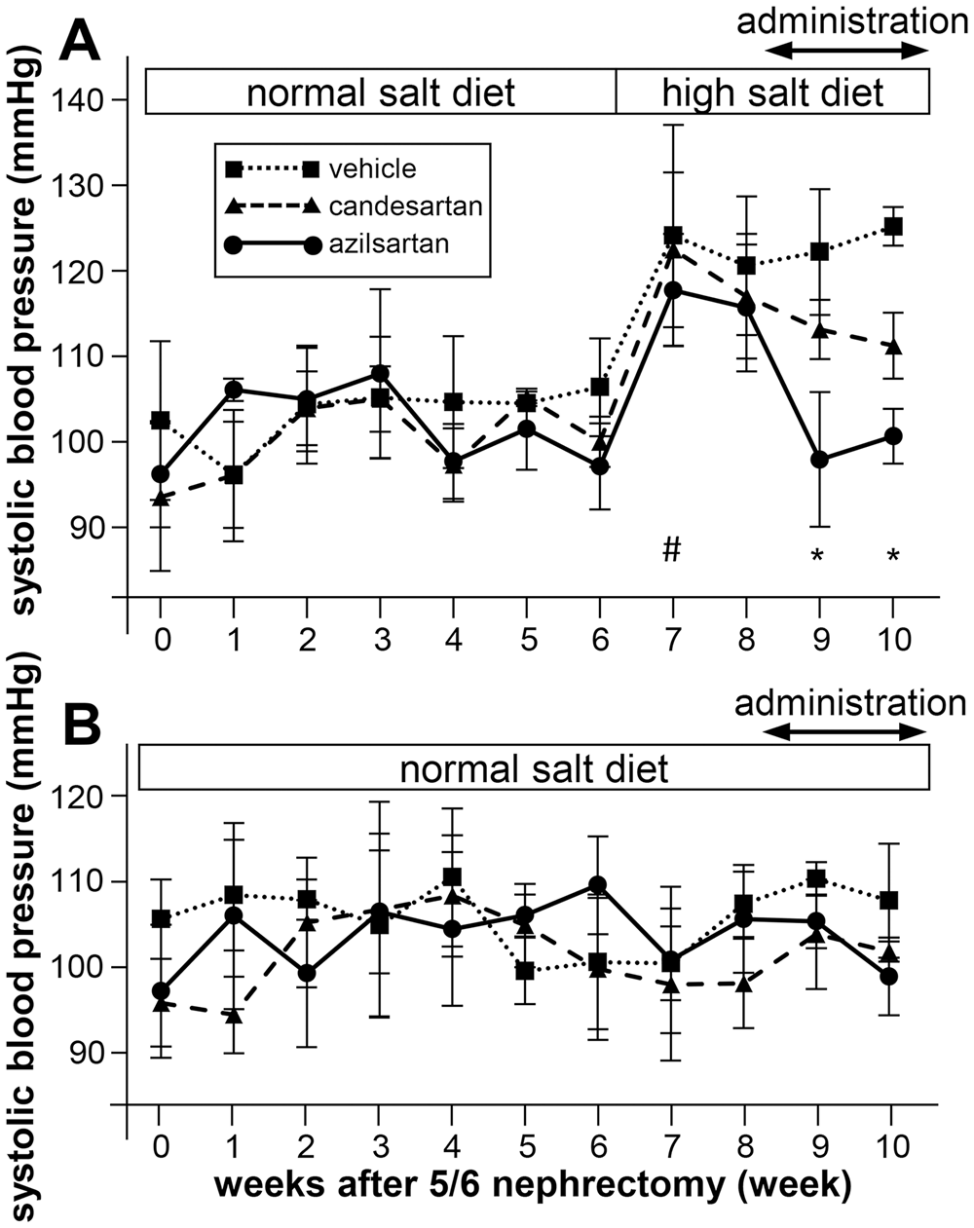
Systolic blood pressure (SBP) after 5/6 nephrectomy (Nx) during the light period in mice fed a high-salt diet and a normal-salt diet. Fig 1A shows changes in SBP of high-salt diet (8% NaCl) groups after 6 weeks, whereas Fig 1B shows SBP changes in normal-salt diet (0.3% NaCl) groups. After 8 weeks, mice were administered drugs or vehicle. In high-salt groups, azilsartan significantly reduced SBP compared with candesartan and vehicle, and candesartan significantly reduced SBP compared with vehicle. In normal-salt diet groups, there were no significant differences in SBP. Data represent mean ± standard deviation. *n* = 3–6 for each group. *P < 0.05 azilsartan *vs*. vehicle, azilsartan *vs*. candesartan, and candesartan *vs*. vehicle. #*P* < 0.05 SBP (week 7) *vs*. SBP (week 6) for each group.

To investigate the effect of azilsartan and candesartan on salt-sensitive hypertension, 5/6 Nx mice were fed a high-salt diet (8% NaCl) and a medium-salt diet (4% NaCl) as a model of salt-sensitive hypertension. Fig 1 shows blood pressure levels during the light period for each week after 5/6 Nx. Although 5/6 Nx mice on a high-salt diet exhibited significantly elevated blood pressure compared with those on a normal-salt diet (117.9 ± 2.5 *vs*. 101.2 ± 1.9 mmHg, *P* < 0.05), treatment with azilsartan or candesartan significantly reduced the blood pressure of mice fed a high-salt diet (azilsartan: 103.1 ± 1.0; candesartan: 111.7 ± 2.7; vehicle: 125.5 ± 2.5 mmHg; *P* < 0.05 azilsartan or candesartan *vs*. vehicle) (Fig 1A). Notably, azilsartan was more effective at reducing blood pressure than candesartan (*P* < 0.05). In contrast to the high-salt diet, neither azilsartan nor candesartan reduced blood pressure compared with the vehicle control (azilsartan: 99.5 ± 3.1; candesartan: 102.4 ± 4.4; vehicle: 108.2 ± 1.4 mmHg; not significant) when the mice were exposed to a normal-salt diet (Fig 1B). In the medium-salt diet paradigm (4% NaCl), there was not aa clear difference blood pressure between the candesartan- and azilsartan-administered mice (S1 Fig).

### Azilsartan improved salt sensitivity in salt-sensitive mice

**Fig 2:**
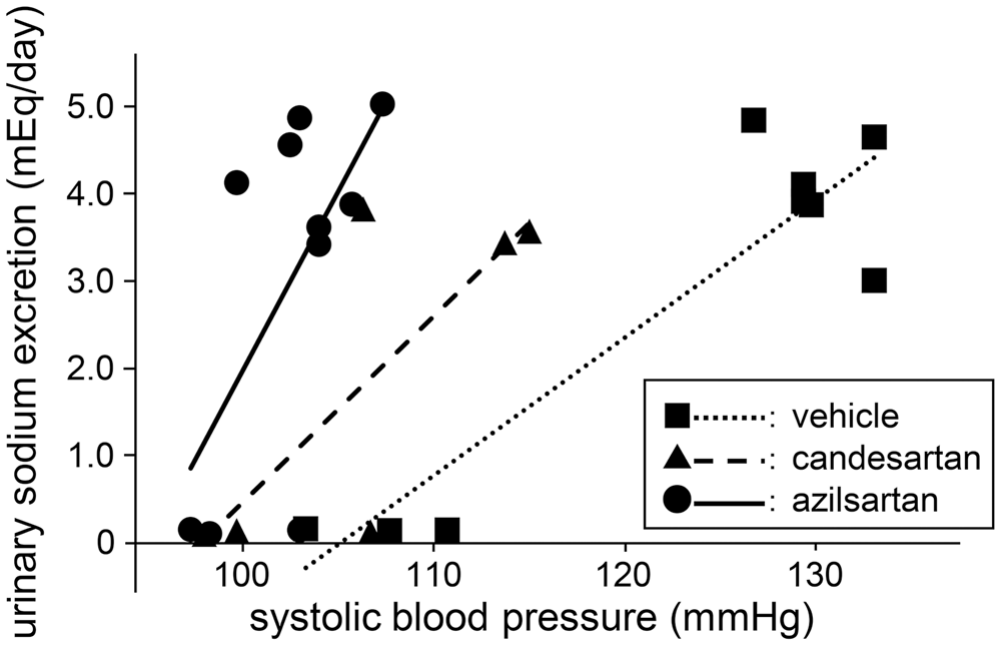
Effect of azilsartan and candesartan on the pressure—natriuresis curve. In the azilsartan group, the curve shifted to the left, with an increased slope compared with the vehicle group. The interaction between urinary excretion of sodium and blood pressure was significant for azilsartan *vs*. vehicle in the linear regression model (*p* for interaction = 0.062). This finding suggested that azilsartan improves salt sensitivity. A similar change in curve features was shown in the candesartan group, but the interaction was not significant for candesartan *vs*. vehicle (*p* for interaction = 0.393; *n* = 5–7 for each group).

To evaluate the effects of azilsartan and candesartan on salt sensitivity, we undertook pressure-natriuresis analyses. At 2 weeks after administration of azilsartan, candesartan, or vehicle, a pressure—natriuresis curve was obtained by plotting urinary excretion of sodium on the ordinate as a function of systolic blood pressure in conjunction with a high-salt or normal-salt diet (Fig 2). Azilsartan treatment increased the slope of the pressure—natriuresis curve compared with the vehicle group, with little effect on the x-intercept. The interaction between urinary excretion of sodium and blood pressure was significant for azilsartan *vs*. vehicle, but not *vs*. candesartan in the linear regression model (*p* for the interaction = 0.062 and 0.242, respectively). This finding suggested that azilsartan improved salt sensitivity. A similar change in curve features was shown in the candesartan group, but the change was small compared with that observed in the azilsartan group. The interaction was not significant for candesartan *vs*. vehicle (*p* for interaction = 0.393). According to data from mice on the medium-salt and normal-salt diets, a similar pressure—natriuresis curve was not observed (S2 Fig). Hence, we analyzed only data from mice on a high-salt and normal-salt diets from this point forward.

### Azilsartan induced natriuresis in the dark period

Physiological data of the high-salt diet groups 2 weeks after treatment are shown in Table 1. No significant differences in blood urea nitrogen or creatinine clearance (index of renal function) were observed among the three groups. Body weight, water intake, and urine volume also did not differ. Serum levels of sodium and U_Na_V did not significantly differ between the three groups, but FENa was significantly higher during the dark period in the azilsartan group compared with the candesartan and vehicle groups. These results showed that azilsartan induced natriuresis during the dark period to a greater extent compared with candesartan and vehicle, and this effect was independent of renal function.

**Table 1:** Physiological data after 2 weeks of treatment.

	Vehicle (*n* = 5)	Candesartan (*n* = 5)	Azilsartan (*n* = 7)
**Serum Na (mEq/l)**	147.8 ± 4.3	148.0 ± 3.8	152.9 ± 4.4
**BUN (mg/dl)**	52.2 ± 15.8	38.5 ± 3.0	47.3 ± 8.0
**Creatinine Clearance (ml/min)**			
**Light Period**	0.162 ± 0.091	0.128 ± 0.054	0.159 ± 0.096
**Dark Period**	0.245 ± 0.092	0.217 ± 0.028	0.191 ± 0.040
**Body Weight (g)**	24.44 ± 2.53	23.68 ± 1.56	25.96 ± 1.80
**Water Intake (ml/d)**	21.7 ± 3.4	23.3 ± 4.3	23.0 ± 5.1
**Urine Volume (ml/12 h)**			
**Light Period**	3.0 ± 1.7	3.3 ± 1.4	3.5 ± 1.6
**Dark Period**	11.5 ± 1.3	13.5 ± 2.6	12.7 ± 2.8
**Urinary Sodium Excretion (mEq/12 h)**			
**Light Period**	0.91 ± 0.41	1.02 ± 0.37	1.00 ± 0.29
**Dark Period**	3.29 ± 0.41	3.48 ± 0.31	3.55 ± 0.54
**Fractional Excretion of Sodium (%)**			
**Light Period**	6.55 ± 0.95	9.90 ± 1.94	11.37 ± 4.13
**Dark Period**	13.85 ± 5.30	14.17 ± 1.42	21.37 ± 3.69*

Fractional excretion of sodium during the dark period was significantly greater only in the azilsartan group compared with the candesartan and vehicle groups.

BUN, blood urea nitrogen. Data represent mean ± standard deviation.

*P < 0.05 azilsartan *vs*. candesartan or vehicle.

### Azilsartan reduced NHE3 protein expression in proximal tubular cells

**Fig 3:**
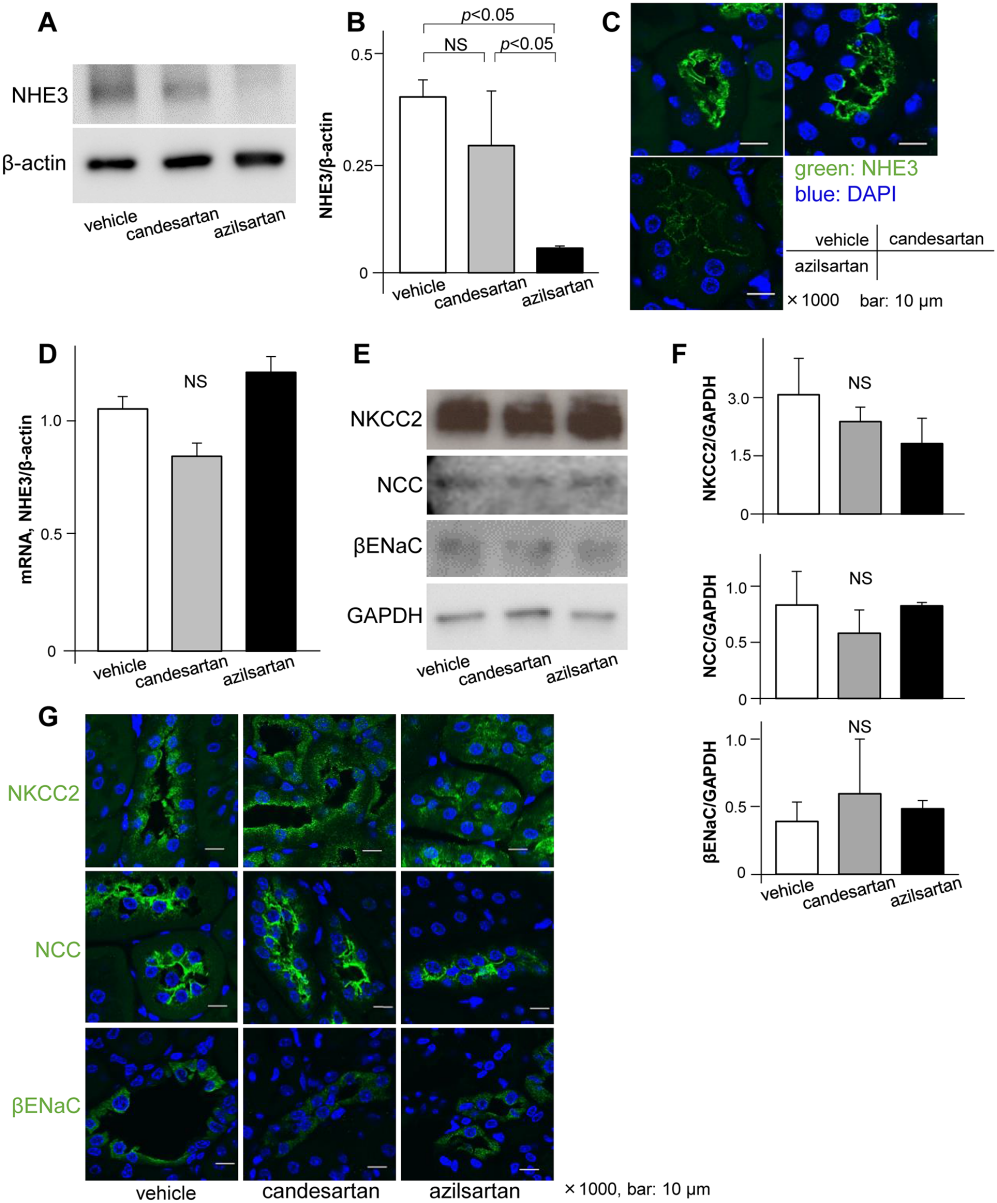
Analyses of sodium transporters *in vivo*. Representative immunoblot analyses of brush border membranes (A) with a summary of all data (B) and immunofluorescence analyses in whole kidneys (C) showed that azilsartan significantly reduced NHE3 protein expression compared with candesartan and vehicle, whereas the NHE3 transcription level remained unchanged in the three groups (D). Immunoblot (E) and immunofluorescence (F) analyses showed that protein expression of downstream sodium transporters in the whole kidney was not reduced by azilsartan compared with candesartan and vehicle. NHE3, Na^+^-H^+^ exchanger-3; NKCC2, Na^+^-K^+^-Cl^−^ cotransporter-2; NCC, Na^+^-Cl^−^cotransporter; βENaC, β epithelial Na^+^ channel; NS, not significant. Data represent mean ± standard deviation. *n* = 3–6 for each group.

**Fig 4:**
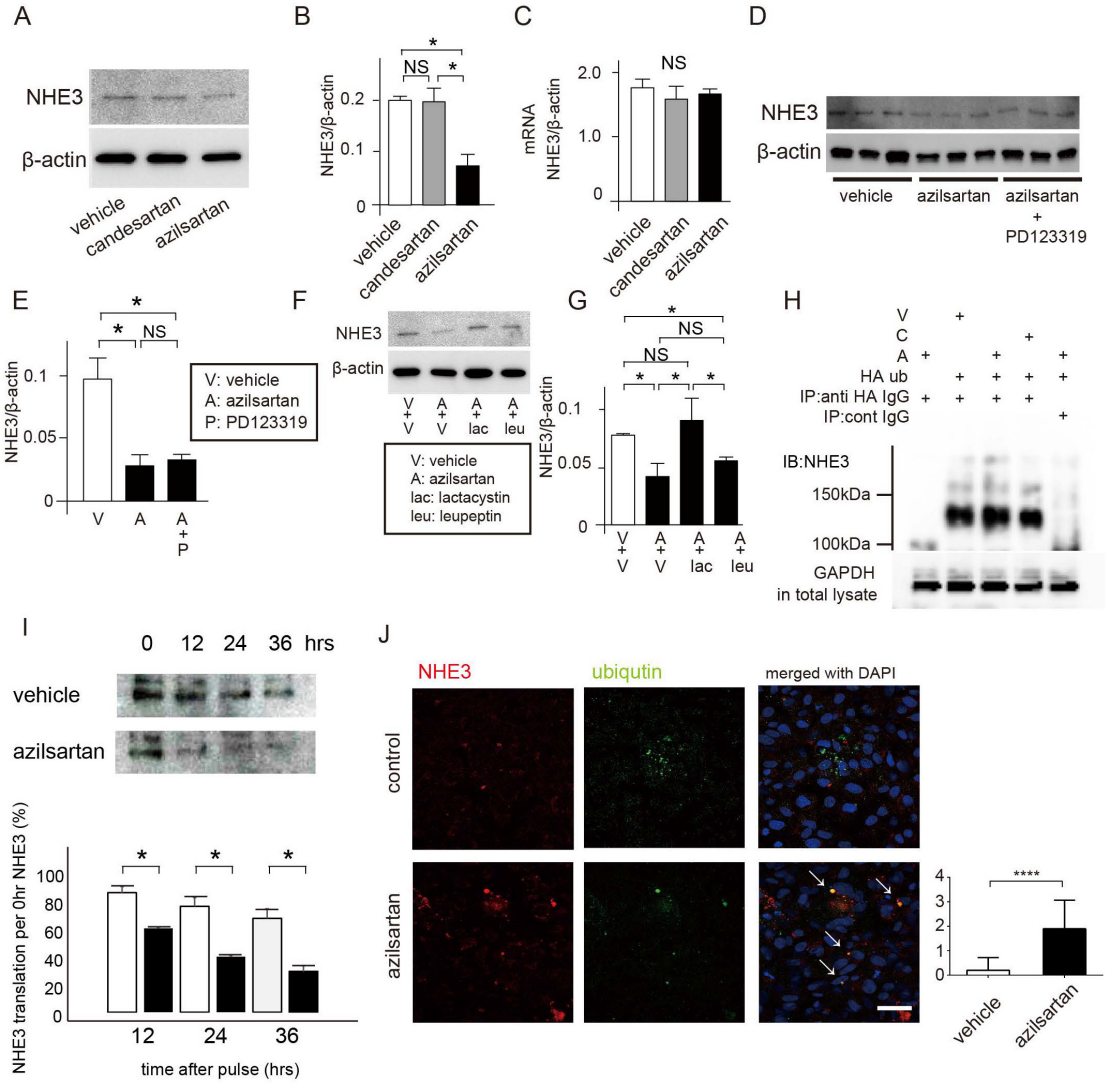
Mechanism of reduction of expression of NHE3 protein with azilsartan. Azilsartan, candesartan, or vehicle was applied to confluent opossum kidney (OK) cells for 24 h. Representative immunoblot (A) with a summary of all data (B) showed that azilsartan significantly reduced NHE3 protein expression compared with candesartan and vehicle, whereas the NHE3 transcription level remained unchanged in the three groups (C). Azilsartan or vehicle was applied to confluent OK cells for 24 h alone or in combination with the angiotensin II receptor type-2 antagonist PD123319. Immunoblot results (D) and a summary of all data (E) showed that azilsartan reduced NHE3 protein expression compared with vehicle treatment, and that PD123319 did not affect these results. Next, azilsartan or vehicle was applied to confluent OK cells for 24 h alone or in combination with lactacystin (proteasomal inhibitor) or leupeptin (lysosomal inhibitor). Representative immunoblot (F) with a summary of all data (G) showed that azilsartan significantly reduced NHE3 protein expression compared with vehicle. Leupeptin did not change this result, but lactacystin cancelled out the effect of azilsartan. Furthermore, OK cells were transiently transfected with HA-Ub; azilsartan, candesartan, or vehicle was subsequently added to the OK cells. Then, HA-Ub was immunoprecipitated with an anti-HA antibody and immunoblotted with anti-NHE3 antibody to detect NHE3 ubiquitination. Representative data indicated that azilsartan induced more ubiquitination than candesartan and vehicle (H). However, similar band patterns were not observed with other sample lanes when control vector-transfected OK cell lysates were immunoprecipitated with an anti-HA antibody, or when HA-Ub expression vector-transfected OK cell lysates were immunoprecipitated with IgG from control mice. (I) Pulse-chase experiments with ^35^S-methionine revealed that azilsartan enhanced NHE3 protein degradation. (J) Immunofluorescent images identified a greater number of NHE3-positive puncta that merged with Ub in OK cells induced by azilsartan. We took 20 images per sample in a blinded fashion and quantified the puncta that co-localized with NHE3 and ubiquitin. Bar = 50 μm. NHE3, Na^+^-H^+^ exchanger-3; AT2R, angiotensin II type-2 receptor; Ub, ubiquitin; NS, not significant. Data represent mean ± standard deviation. *n* = 3–6 for each group.

To reveal the mechanism of improved natriuresis by azilsartan, we evaluated expression of the major tubular sodium transporter in proximal tubules (NHE3), NKCC2 in the loop of Henle, NCC in distal tubules, and ENaC in the collecting ducts in mice on a high-salt diet (Fig 3, S3 Fig). Western blot and immunofluorescence studies showed that azilsartan reduced NHE3 protein expression compared with candesartan and vehicle (Fig 3A–3C, S3A Fig); however, azilsartan did not affect NHE3 transcription levels (Fig 3D). Azilsartan did not affect protein expression of NKCC2, NCC, or ENaC (which are all downstream sodium transporters) compared with candesartan or vehicle (Fig 3E–3G, S3B–S3D Fig). These data suggest that azilsartan caused natriuresis by reducing NHE3 protein expression in proximal tubules without altering NHE3 gene expression. We confirmed the effect of azilsartan on NHE3 protein expression using cultured OK proximal tubular cells. As for the *in vivo* results, azilsartan treatment reduced NHE3 expression at the protein level compared with candesartan and vehicle (Fig 4A and 4B), but not at the transcriptional level (Fig 4C). These effects were not cancelled out by the AT2R antagonist PD123319 (Fig 4D and 4E). These results suggest that azilsartan accelerated NHE3 protein degradation.

### Azilsartan promoted NHE3 protein degradation *via* an ubiquitin—proteasomal pathway

To investigate the mechanism responsible for azilsartan-induced reduction in NHE3 protein expression, we investigated which pathway (proteasomal or lysosomal) was involved in NHE3 degradation. Azilsartan- or vehicle-treated cultured OK cells were administered lactacystin (proteasomal inhibitor), leupeptin (lysosomal inhibitor), or vehicle. Leupeptin did not alter the effect of azilsartan on NHE3 expression, but lactacystin blocked it (Fig 4F and 4G). These data suggest that azilsartan accelerated NHE3 degradation *via* a proteasomal pathway. Ubiquitination plays a major part in proteasomal degradation; therefore, we examined whether NHE3 ubiquitination was accelerated by azilsartan. OK cells, transiently transfected with HA-Ub, were treated with azilsartan, candesartan, or vehicle. Thereafter, HA-Ub was immunoprecipitated with anti-HA antibody and immunoblotted with an anti-opossum NHE3 antibody to detect NHE3 ubiquitination. Azilsartan increased NHE3 ubiquitination compared with candesartan and vehicle (Fig 4H), suggesting that azilsartan induces NHE3 protein degradation *via* an ubiquitin—proteasomal pathway. In accordance with these observations, pulse-chase experiments revealed that azilsartan induced more degradation of ^35^S-labeled NHE3 than vehicle. Furthermore, immunofluorescence images identified more NHE3- and ubiquitin-positive puncta in OK cells induced by azilsartan than by vehicle.

## Discussion

Results from the present study demonstrated that the novel and potent ARB azilsartan improved salt-sensitive hypertension by reducing NHE3 protein expression *via* degradation through an ubiquitin—proteasomal pathway. This finding is surprising, because ARBs have been reported to potentiate salt sensitivity [20, 21]. However, a recent study [24, 35] using azilsartan in hypertensive patients revealed that azilsartan improves the circadian rhythm of blood pressure, suggesting that this ARB can alter sodium handling in the kidney and modify salt sensitivity. We sought to determine how azilsartan changes sodium handling in the kidney using a salt-sensitive mouse model of hypertension.

Like other ARBs, azilsartan is highly selective for the angiotensin II type-1 receptor (AT1R) and has ≥10,000-fold greater affinity for AT1R compared with AT2R. Azilsartan also functions as an inverse agonist and inhibits AT1R signaling [36]. Azilsartan has a unique side chain, 5-oxo-1,2,4-oxadiazole, in place of the tetrazole ring in candesartan. This unique azilsartan moiety may be responsible for the strong antagonistic activity and higher affinity for AT1R compared with candesartan and other ARBs [37, 38].

First, we showed that azilsartan (not candesartan) improved salt-sensitive hypertension in 5/6 Nx mice. In our study, the 5/6 Nx mice exhibited increased blood pressure on a high-salt diet compared with a normal-salt diet, suggesting that the 5/6 Nx mice developed salt-sensitive hypertension. FENa significantly increased during the dark period in the azilsartan group compared with candesartan and vehicle groups, but U_Na_V levels did not increase. However, it has been reported that 4–5 days is sufficient for sodium balance and arterial pressure to reach a steady state on a fixed sodium intake [39]. We did not detail food intake, but mice were allowed to eat *ad libitum*. The body weights among groups were not different, suggesting similar food intake. Our study analyzed urine after 2 weeks of treatment, and the results showed a steady state of U_Na_V. Thus, FENa (not U_Na_V) represented natriuresis.

Analyses of renal tubular sodium transporters showed that azilsartan reduced NHE3 protein expression (but not at the transcriptional level) and did not reduce protein expression of the downstream transporters NKCC2, NCC, or ENaC. NHE3 is an integral protein expressed in the brush border membranes of epithelial cells in the small intestine, colon, and kidney, where it conducts absorption of Na^+^ and HCO_3_^−^ [40, 41]. NHE3 expression is decreased and natriuresis increased in proximal tubule-specific angiotensin II type-1a receptor (AT1aR) knockout mice [42]. This finding suggests that decreased NHE3 expression resulted in natriuresis. Angiotensin II has been reported to increase NHE3 expression in the chronic phase [43–47] and NHE3 expression is decreased in proximal-tubule-specific AT1aR KO mice [42]. This finding indicates that azilsartan (which strongly blocks the effect of angiotensin II) reduced NHE3 expression. Brooks and colleagues reported that gamma-ENaC expression increases in NHE3 knockout mice [48]. However, in our study, expression of the downstream sodium transporter remained unchanged in the three groups. This phenomenon could be due to reduced ENaC levels by ARBs as a result of decreased aldosterone expression [49].

Crosstalk exists between AT1R and AT2R, and they have counter-regulatory functions in several systems, especially the cardiovascular system [50]. Increasing evidence suggests that AT1R is responsible for the classical actions of angiotensin II (e.g., induction of vasoconstriction and cardiovascular hypertrophy), whereas AT2R is directly implicated in vasodilation and anti-growth effects [51]. However, in our study, the AT2R antagonist PD123319 did not alter the effect of azilsartan on NHE3 protein expression. This finding suggested that azilsartan increased NHE3 ubiquitination *via* AT1R blockade, not by counter-stimulation of the AT2R.

Ubiquitination is a post-translational modification that conjugates ubiquitin to lysine residues in target proteins and governs their intracellular fate. Decreased NHE3 protein expression has been shown to take place *via* an ubiquitin—proteasomal pathway [32, 52]. Di Sole *et* al. [52] reported reduced NHE3 protein and transcript expression in acute ischemia—reperfusion injury, and that this reduction was mediated *via* an ubiquitin—proteasomal pathway. They inferred that a certain transferable factor(s) mediated this decrease, and that the factor(s) were likely to be proteins or proteolipid complexes. Hu *et* al. [32] reported that chronic administration of dopamine decreases NHE3 translation and increases NHE3 degradation *via* an ubiquitin—proteasomal pathway. In the present study, NHE3 was also regulated through *cis*-acting sequences of NHE3 mRNA 5′-UTR. Recently, Armando *et* al. [53] reported that ubiquitin-specific peptidase (USP) 48 was associated with NHE3 degradation *via* dopamine. That is, the dopamine D3 receptor inhibits USP48 expression, which promotes NHE3 degradation. We identified an ubiquitin—proteasomal pathway that was responsible for the azilsartan-induced NHE3 degradation. The dopamine D3 receptor—USP48 pathway is thought to be associated with decreased NHE3 expression by azilsartan, because the AT1R forms a complex with the dopamine D3 receptor [54]. Thus, we hypothesize that NHE3 undergoes de-ubiquitination by USP48 in the basal state, which is inhibited by the D3 receptor. The D3 receptor is also inhibited by AT1R, as long as angiotensin-II stimuli are present. When azilsartan blocks AT1R, the D3 receptor inhibits USP48 expression. NHE3 does not undergo de-ubiquitination by USP48, so NHE3 begins proteasomal degradation. As a result, NHE3 function decreases, which results in natriuresis and improved salt sensitivity.

We could reproduce *in vivo* NHE3 data in an *in vitro* OK cell culture system using a normal sodium concentration. However, cell-culture findings with azilsartan at a normal sodium concentration do not necessarily corroborate animal studies involving treatment with high-salt diets and azilsartan.

## Conclusions

We found that the ARB azilsartan improved salt sensitivity, possibly by reducing NHE3 levels *via* increased ubiquitin—proteasomal degradation. This novel mechanism of action could lead to new mechanistic insights into salt sensitivity and, thus, novel therapeutic options for cardiovascular diseases.

## Supporting Information

S1 Fig Systolic blood pressure (SBP) during the light period in mice exposed to 5/6 nephrectomy (Nx) and a medium-salt or normal-salt diet. This figure shows SBP changes in high-salt diet (4% NaCl) groups after 6 weeks. A medium-salt diet induces increased blood pressure in the three groups, but 10 weeks after 5/6 nephrectomy, there is no a clear difference in blood pressure in mice treated with candesartan or azilsartan. Data represent mean ± standard deviation. *n* = 3–6 for each group. NS azilsartan *vs*. vehicle, NS azilsartan *vs*. candesartan, and * *P* < 0.05 candesartan *vs*. vehicle. #*P* < 0.05 SBP (week 8) *vs*. SBP (week 6) for each group.
(TIF)

S2 Fig Pressure—natriuresis curve of mice treated with 4% NaCl or 0.3% NaCl. Significant interactions between urinary excretion of sodium and blood pressure are not observed for azilsartan *vs*. vehicle.
(TIF)

S3 Fig Immunofluorescence images at low magnification (×600) for NHE3 (A), NKCC2 (B), NCC (C), and β-Enac (D) in mice administered vehicle, candesartan, or azilsartan. (TIF)

S1 Table Characterization of antibodies specific to sodium transporters used in the present study. (DOCX)
