# Supplementary figures and images for: Azilsartan Improves Salt Sensitivity by Modulating the Proximal Tubular Na+-H+ Exchanger-3 in Mice
Source: PLoS One. 2016 Jan 25;11(1):e0147786. doi: 10.1371/journal.pone.0147786 (PMC4725961; doi:10.1371/journal.pone.0147786)

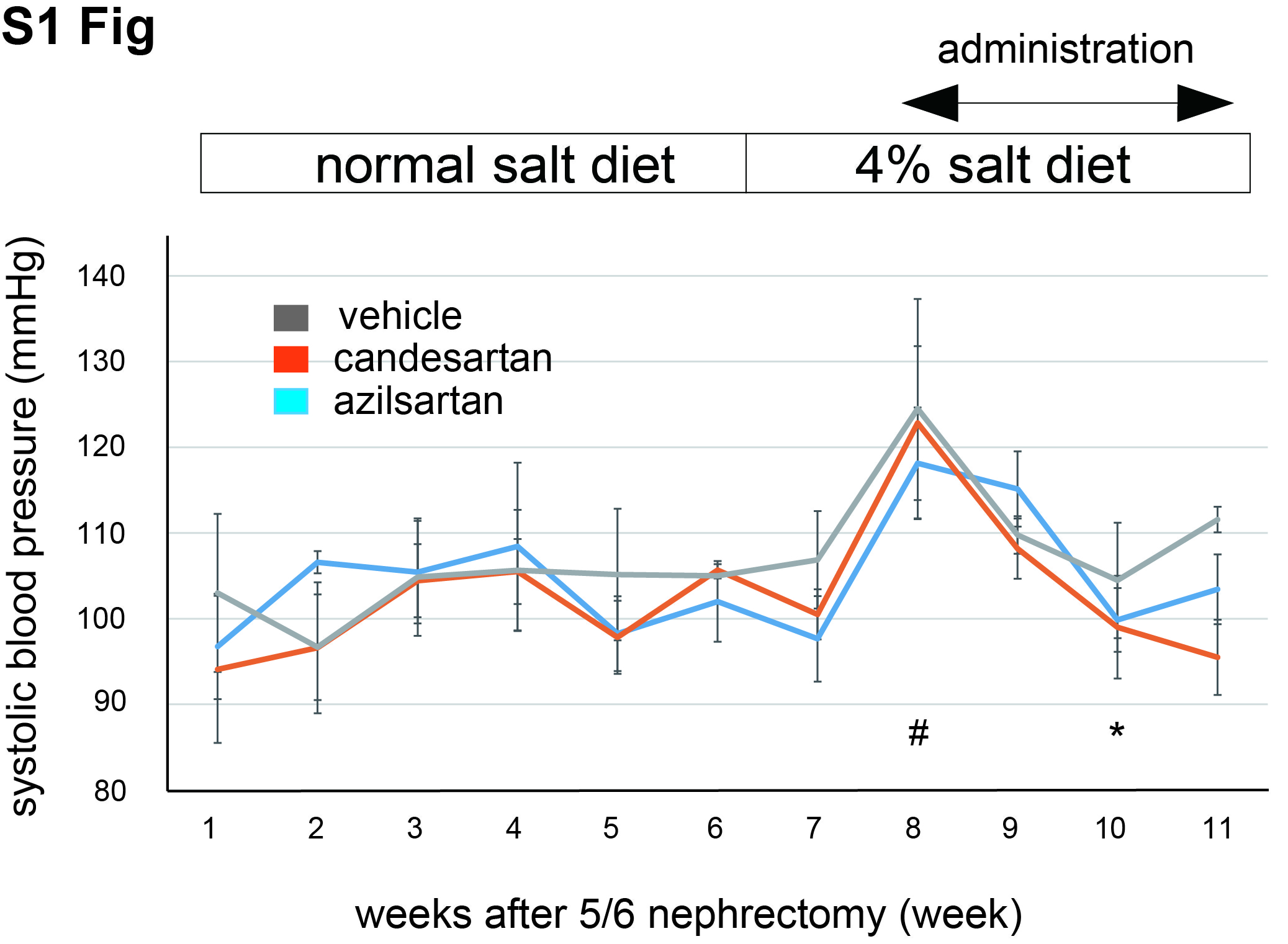

Supplement: S1 Fig — This figure shows SBP changes in high-salt diet (4% NaCl) groups after 6 weeks. A medium-salt diet induces increased blood pressure in the three groups, but 10 weeks after 5/6 nephrectomy, there is no a clear difference in blood pressure in mice treated with candesartan or azilsartan. Data represent mean ± standard deviation. n = 3–6 for each group. NS azilsartan vs. vehicle, NS azilsartan vs. candesartan, and * P < 0.05 candesartan vs. vehicle. #P < 0.05 SBP (week 8) vs. SBP (week 6) for each group. (TIF) [file pone.0147786.s001.tif]

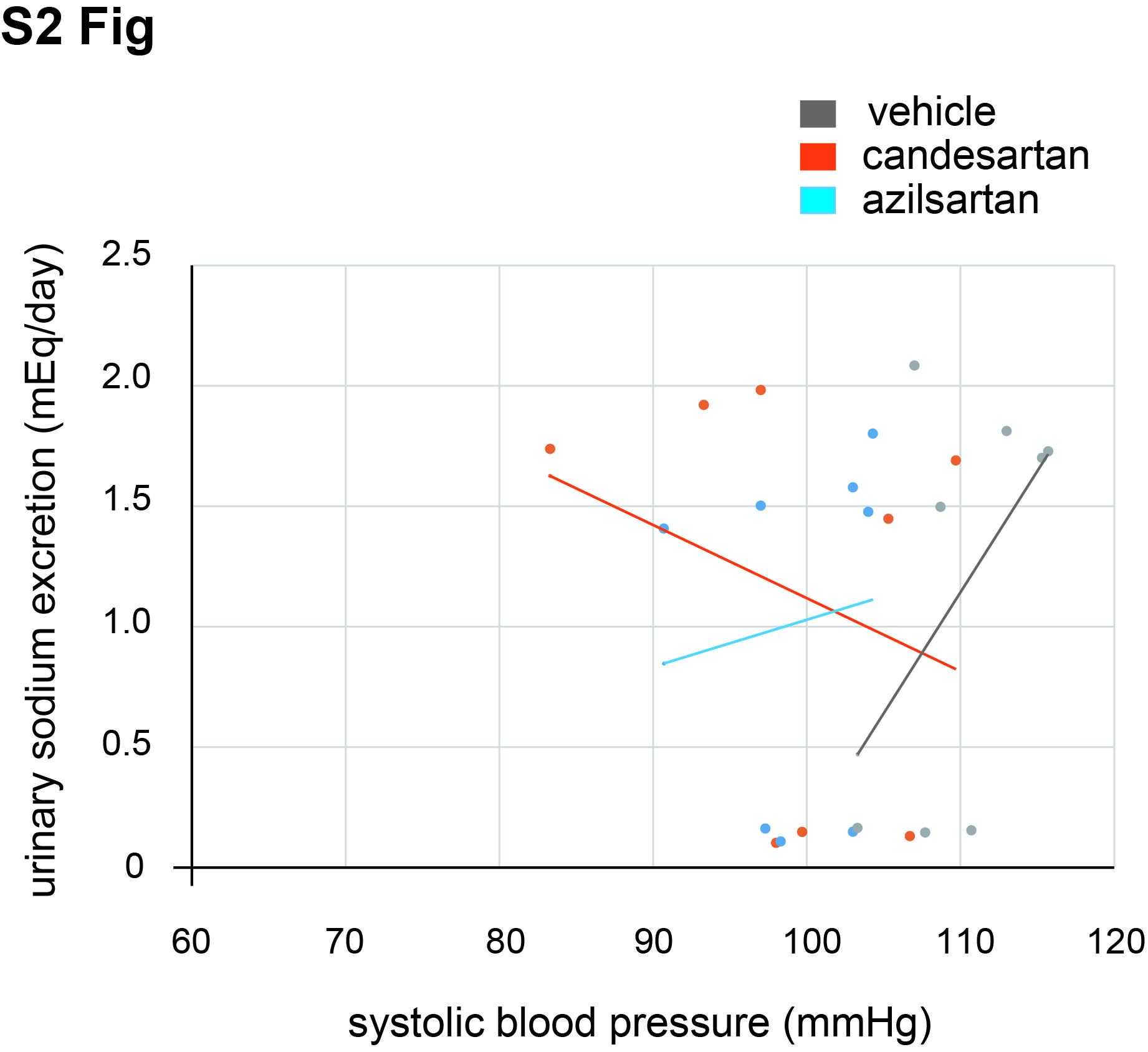

Supplement: S2 Fig — Significant interactions between urinary excretion of sodium and blood pressure are not observed for azilsartan vs. vehicle. (TIF) [file pone.0147786.s002.tif]

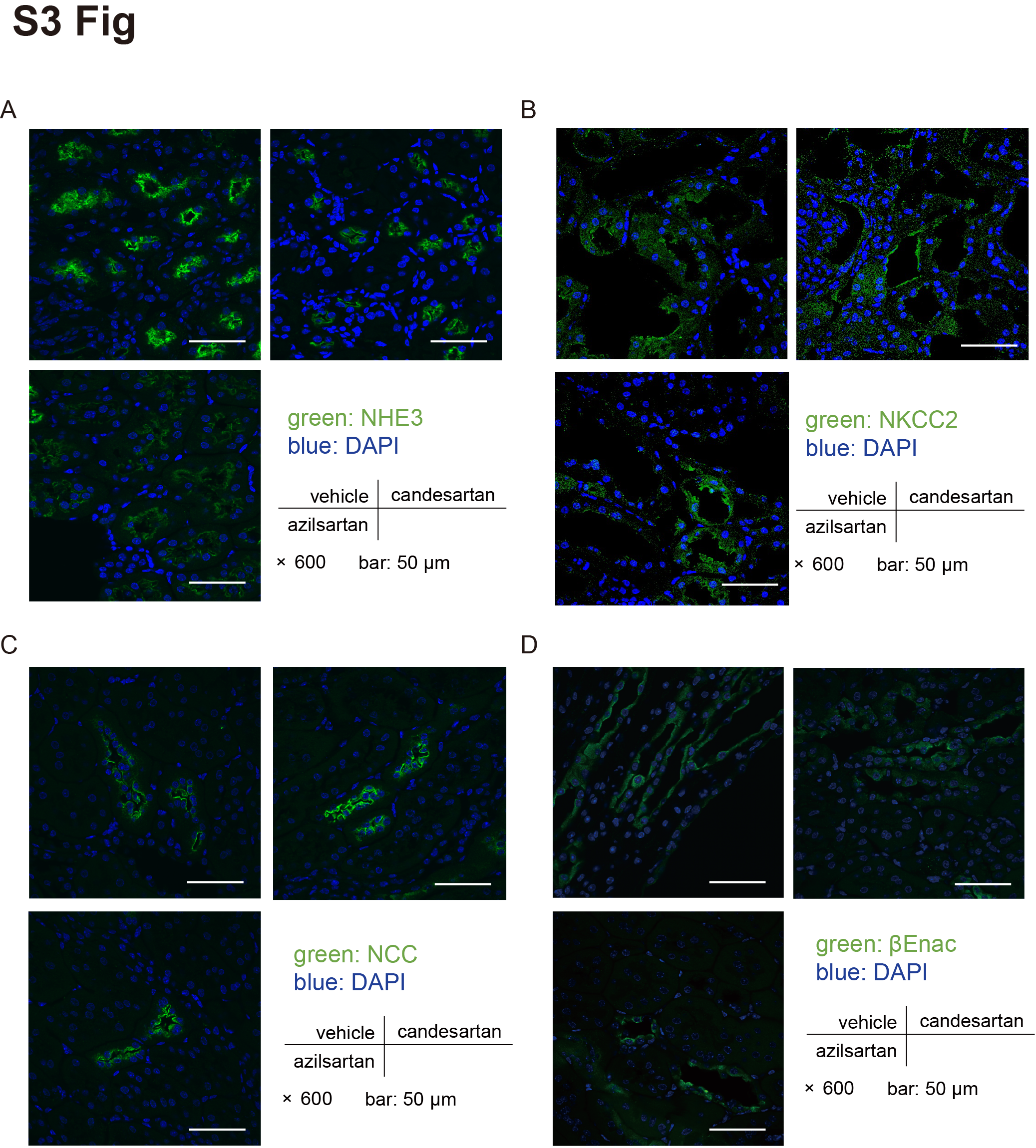

Supplement: S3 Fig — (TIF) [file pone.0147786.s003.tif]
