# Supplementary material for: Azilsartan Improves Salt Sensitivity by Modulating the Proximal Tubular Na+-H+ Exchanger-3 in Mice
Source: PLoS One. 2016 Jan 25;11(1):e0147786. doi: 10.1371/journal.pone.0147786 (PMC4725961; doi:10.1371/journal.pone.0147786)
Supplement: S1 Table — (DOCX) [file pone.0147786.s004.docx]

S1 Table

| Sodium transporter antibody names | Name of provider | Validation |
| --- | --- | --- |
| Rabbit polyclonal anti-NHE3 (SAB5200102,  Lot; 1006) | Sigma-Aldrich | The provider claims that the antibody is suitable for immunohistochemistry of mouse tissue (http://www.sigmaaldrich.com/catalog/product/sigma/av41388?lang=ja&region=JP&cm_sp=Insite-_-prodRecCold_xorders-_-prodRecCold2-1). We validated the antibody in immunohistochemistry of mouse tissue. |
| Mouse monoclonal anti-NHE3 (clone 3H3) | Dr. Peter Aronson (Yale University) | The antibody was validated in western blot analysis of OK cell NHE3 [[1](#_ENREF_1)] and immunocytochemistry of OK cell NHE3 [[2](#_ENREF_2)]. We validated the antibody in western blot analysis of NHE3 using mouse tissue lysates. |
| Rabbit polyclonal anti-NKCC2 (AV41388, Lot; QC12072) | Sigma-Aldrich | The provider claims that it is suitable for western blot analysis and immunohistochemistry in mouse tissue (<http://www.sigmaaldrich.com/catalog/product/sigma/>av41388?lang=ja&region=JP).  We also validated it by western blot analysis and immunohistochemistry. |
| Rabbit polyclonal anti-NCC (AB3553, Lot; 2274029) | Merck Millipore | The provider claims that they validated the antibody by western blot analysis and immunohistochemistry in mouse tissue  (<http://www.merckmillipore.com/JP/ja/product/>  Anti-Thiazide-Sensitive-NaCl-Cotransporter-Antibody,MM_NF-AB3553?bd=1#anchor_  COA), and this antibody was also previously verified [[3](#_ENREF_3)]. We also validated it by western blot analysis and immunohistochemistry. |
| Rabbit polyclonal anti-βEnac | Dr. Carolyn Ecelbarger | The antibody was previously validated [[4](#_ENREF_4)]. We also validated it by western blot analysis and immunohistochemistry. |

1. Girardi AC, Knauf F, Demuth HU, Aronson PS, Role of dipeptidyl peptidase IV in regulating activity of Na+/H+ exchanger isoform NHE3 in proximal tubule cells. Am J Physiol Cell Physiol 2004;287: C1238-1245.

2. He P, Klein J, Yun CC, Activation of Na+/H+ exchanger NHE3 by angiotensin II is mediated by inositol 1,4,5-triphosphate (IP3) receptor-binding protein released with IP3 (IRBIT) and Ca2+/calmodulin-dependent protein kinase II. J Biol Chem 2010;285: 27869-27878.

3. Nijenhuis T, Hoenderop JG, Loffing J, van der Kemp AW, van Os CH, et al., Thiazide-induced hypocalciuria is accompanied by a decreased expression of Ca2+ transport proteins in kidney. Kidney Int 2003;64: 555-564.

4. Masilamani S, Kim GH, Mitchell C, Wade JB, Knepper MA, Aldosterone-mediated regulation of ENaC alpha, beta, and gamma subunit proteins in rat kidney. J Clin Invest 1999;104: R19-23.
